# Painful pink papules and nodules presenting in a Blaskoid distribution

**DOI:** 10.1016/j.jdcr.2024.10.014

**Published:** 2024-11-02

**Authors:** Megan Hauptman, Mio Nakamura, Michael Goldfarb

**Affiliations:** Department of Dermatology, University of Michigan, Ann Arbor, Michigan

**Keywords:** medical dermatology, mosaicism, oncogenes

## Case presentation

**Fig 1:**
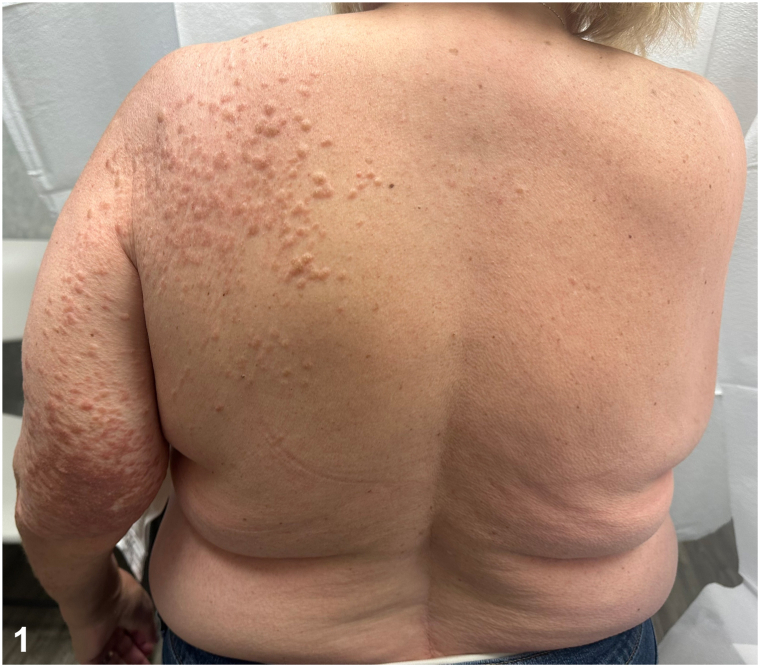

**Fig 2:**
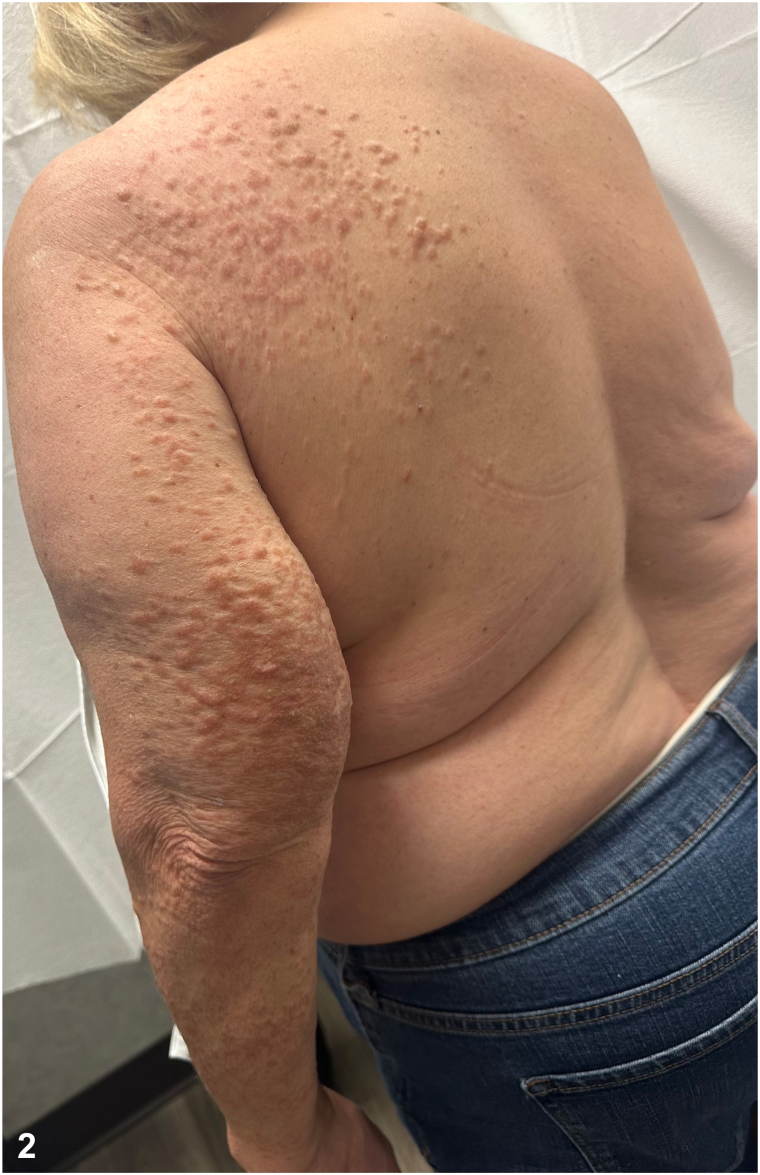

A 66-year-old female presented for evaluation of skin lesions on the left shoulder and arm. They first developed on her left shoulder 40 years prior and since grew and spread to her arm. She described them as “hot pokers” that are tender to touch and cold temperatures. Medical history was notable for uterine leiomyomas resulting in a hysterectomy at 35 years old. Physical exam showed dull pink papules and plaques in Blaskoid distribution from the left upper back to wrist (Figs 1 and 2). Lesional biopsy stained positive for anti-S-(2-succino)-cysteine (2SC) and negative for fumarate hydratase (FH). Genetic testing revealed a germline FH mutation.

**Question 1:** **What is the most likely underlying diagnosis?**

A. Interlacing bundles of smooth muscle cells in the upper dermis
B. Solid sheets of glomus cells around small blood vessels
C. Atypical neoplastic cells arranged in small nests and cords surrounded by fibrosis
D. Zonal pattern composed of cellular areas with nuclear palisading and a hypocellular component
E. Polygonal cells with bland nuclei, abundant cytoplasm, and fine eosinophilic cytoplasmic granules

**Answers:**

A. Interlacing bundles of smooth muscle cells in the upper dermis – Correct. Piloleiomyomas originate from the arrector pili muscle of the pilosebaceous unit and occur as solitary or multiple lesions. Leiomyomas are painful, hyperpigmented firm reddish-brown papules and nodules on the trunk and extremities.
B. Solid sheets of glomus cells around small blood vessels – Incorrect. Glomus tumors arise from the glomus body or Sucquet-Hoyer canals. Glomus tumors are painful, 1-2 cm reddish-blue papule or nodule on or around a nailbed.
C. Atypical neoplastic cells arranged in small nests and cords surrounded by fibrosis – Incorrect. Cutaneous metastases present as firm, mobile, skin colored to red round or oval nonpainful nodules.
D. Zonal pattern composed of cellular areas with nuclear palisading and a hypocellular component – Incorrect. Neurilemmomas are benign peripheral nerve sheath tumors composed of Schwann cells. Neurilemmoma present as a palpable mass that can be mobilized in the plane orthogonal to the axis of the affected nerve. They are associated with paresthesia throughout the distribution of the affected nerve.
E. Polygonal cells with bland nuclei, abundant cytoplasm, and fine eosinophilic cytoplasmic granules – Incorrect. Granular cell tumors present as a slow-growing, solitary, occasionally painful and pruritic 1-3 cm skin-cored to brown-red nodules most commonly on the head and neck.

**Question 2:** **What histological features did the lesional biopsy most likely demonstrate?**

A. Hereditary leiomyomatosis and renal cell cancer (HLRCC)
B. Neufibromatosis type I
C. Metastatic tumors in a dermatomal distribution
D. Familial glomuvenous malformation
E. Brooke-Spiegler syndrome

**Answers:**

A. Hereditary leiomyomatosis and renal cell cancer (HLRCC) – Correct. This case exemplifies HLRCC presenting with the unique feature of unilateral type 2 mosaicism. HLRCC is a rare autosomal dominant (AD) condition characterized by cutaneous leiomyomas, uterine leiomyomas, and type 2 papillary renal cell carcinoma.^1^ HLRCC is caused by a germline heterozygous mutation in the FH gene. High levels of 2SC accumulate because fumarate can nonenzymatically react with cysteine sulfhydryl groups to form 2SC. Cutaneous leiomyomas can present in a mosaic pattern represented by the lines of Blashko.^2^ Type 1 mosaicism results from a de novo postzygotic mutation during embryogenesis of one allele resulting in a mosaic pattern. Type 2 mosaicism results from a deleterious germline mutation resulting in a postzygotic mutation. The resulting “second hit” results in loss of heterozygosity and early-onset of more severe skin tumors with segmental distribution.
B. Neufibromatosis type I – Incorrect. Neurofibromatosis 1 is an AD disorder associated with multiple neurofibromas, lisch nodules, café-au-lait macules, and freckling in skin folds.
C. Metastatic tumors in a dermatomal distribution – Incorrect. Metastatic tumors may present as dull pink papules and plaques; however, staining positive for 2SC and negative for FH is specific to HLRCC.
D. Familial glomuvenous malformation – Incorrect. Familial glomuvenous malformation is an AD disorder resulting in segmental tender, soft, pink-red to blue nodules due to abnormal proliferation of the vasculature.
E. Brooke-Spiegler syndrome – Incorrect. Brooke-Spiegler syndrome is an AD disorder resulting in cylindromas, trichoepitheliomas, and spiradenomas.

**Question 3: What is the recommended monitoring for this syndrome?**

A. Yearly abdominal magnetic resonance imagings (MRIs) and skin exams
B. Yearly renal ultrasounds and FH genetic testing in first- and second-degree relatives
C. Yearly abdominal computed tomographies (CTs)
D. Annual blood urea nitrogen (BUN)/creatinine (Cr) and urinalysis (UA)
E. No screening necessary

**Answers:**

A. Yearly abdominal MRIs and skin exams – Correct. Approximately 10% to 15% of HLRCC cases are associated with type II papillary renal cell carcinoma.^3^ Because the case presented herein with cutaneous mosaicism has a germline FH mutation, she has the same risk of developing type II papillary renal cell carcinoma as an individual with HLRCC who presents without cutaneous mosaicism. Current guidelines recommend annual contrast-enhanced MRI of the kidneys. All patients should be followed by a dermatologist for annual skin exams, as leiomyomas can turn into leiomyosarcoma in approximately 1% of cases.^4^
B. Yearly renal ultrasounds and FH genetic testing in first- and second-degree relatives – Incorrect. Contrast-enhanced MRIs are more sensitive than renal ultrasounds for type II papillary renal cell carcinoma screening. FH genetic testing in first- and second-degree relatives may be considered so appropriate screenings can be implemented to monitor for associated malignancies.
C. Yearly abdominal CTs – Incorrect. Current guidelines recommend annual contrast-enhanced MRI of kidneys to screen for type II papillary renal cell carcinoma.
D. Annual BUN/Cr and UA – Incorrect. Current guidelines recommend annual contrast-enhanced MRI to screen for type II papillary renal cell carcinoma.
E. No screening necessary – Incorrect. Individuals with HLRCC are at high risk of developing renal cell carcinoma, so annual screenings with a contrast-enhanced MRI of the kidneys are recommended.

## Conflicts of interest

None disclosed.
